# Estimation of genetic variation in vitiligo associated genes: Population genomics perspective

**DOI:** 10.1186/s12863-024-01254-6

**Published:** 2024-07-26

**Authors:** Neeraj Bharti, Ruma Banerjee, Archana Achalare, Sunitha Manjari Kasibhatla, Rajendra Joshi

**Affiliations:** HPC-Medical and Bioinformatics Applications Group, Centre for Development of Advanced Computing, Innovation Park, Pashan, Pune, 411008 Maharashtra India

**Keywords:** Vitiligo, Population genomics, 1KGP, Genetic risk, SNP, Disease prevalence, Risk allele

## Abstract

**Background:**

Vitiligo is an auto-immune progressive depigmentation disorder of the skin due to loss of melanocytes. Genetic risk is one of the important factors for development of vitiligo. Preponderance of vitiligo in certain ethnicities is known which can be analysed by understanding the distribution of allele frequencies across normal populations. Earlier GWAS identified 108 risk alleles for vitiligo in Europeans and East Asians. In this study, 64 of these risk alleles were used for analysing their enrichment and depletion across populations (1000 Genomes Project and IndiGen) with reference to 1000 Genomes dataset. Genetic risk scores were calculated and Fisher’s exact test was performed to understand statistical significance of their variation in each population with respect to 1000 Genomes dataset as reference. In addition to SNPs reported in GWAS, significant variation in allele frequencies of 1079 vitiligo-related genes were also analysed. Two-tailed Chi-square test and Bonferroni’s multiple adjustment values along with fixation index (≥ 0.5) and minimum allele frequency (≥ 0.05) were calculated and used to prioritise the variants based on pairwise comparison across populations.

**Results:**

Risk alleles rs1043101 and rs10768122 belong to 3 prime UTR of glutamate receptor gene *SLC1A2* are found to be highly enriched in the South Asian population when compared with the ‘*global normal*’ population. Intron variant rs4766578 (*ATXN2*) was found to be deleted in SAS, EAS and AFR and enriched in EUR and AMR1. This risk allele is found to be under positive selection in SAS, AMR1 and EUR. From the ancillary vitiligo gene list, nonsynonymous variant rs16891982 was found to be enriched in the European and the Admixed American populations and depleted in all others. rs2279238 and rs11039155 belonging to the *LXR-α* gene involved in regulation of metalloproteinase 2 and 9 (melanocyte precursors) were found to be associated with vitiligo in the North Indian population (in earlier study).

**Conclusion:**

The differential enrichment/depletion profile of the risk alleles provides insight into the underlying inter-population variations. This would provide clues towards prioritisation of SNPs associated with vitiligo thereby elucidating its preponderance in different ethnic groups.

**Supplementary Information:**

The online version contains supplementary material available at 10.1186/s12863-024-01254-6.

## Background

Vitiligo is an acquired pigmentation disorder characterised by the loss of functional melanocytes resulting in the development of depigmented macules and patches on the skin [1]. It affects ∼ 0.5-2% of the world’s population and is observed to have the highest prevalence of ∼ 8.8% in few states of India [2–4]. The exact etiology of vitiligo remains unknown and involves multiple factors such as genetic, immunological, and environmental [5]. The autoimmune nature of this polygenic disorder, combined with involvement of multiple susceptibility loci with different degrees of penetrance cannot be explained by Mendelian genetics [6]. Vitiligo is found to be commonly associated with other autoimmune diseases viz., atopic dermatitis, alopecia areata and psoriasis [7]. Genome wide association studies (GWAS) have the potential to identify multiple loci associated with such complex diseases [8]. Polygenic risk scores aggregate the cumulative effects of multiple identified variants from GWAS responsible for a particular disease in a sampled population, and estimate an individual’s genetic predisposition towards a complex trait [9, 10]. Genetic risk score calculates the probability of an individual for predisposition towards developing the disease [11].

Several susceptibility genes associated with vitiligo, including HLA class I and II genes, *NLRP1*, *PTPN22*, and *FOXP3* have been identified using cohort-based GWAS [6]. Studies of allele frequency distribution of SNPs belonging to genes related to pigmentation, viz., *HERC2*, *MC1R* have also been found to be associated with susceptibility to vitiligo [12].

Majority of GWAS studies for vitiligo are limited to Caucasian and East Asian populations [6, 13–15]. The risk alleles identified in GWA studies for diseases such as age-related macular disorders, obesity and psoriasis have been used to probe prevalence in normal individuals belonging to other population groups [16–19]. Hence, genetic variation of risk alleles across populations can be one of the factors to understand disease prevalence [20].

National Human Genome Research Institute-European Bioinformatics Institute (NHGRI-EBI) GWAS catalogue lists 108 loci to be associated with the prevalence of vitiligo [21]. In this study we aim to investigate the association of genetic risk scores of vitiligo risk alleles in normal individuals across different ethnic populations. As most GWAS pertain to European and East Asian ethnicities, the objective of the current work is to estimate genetic variation of these vitiligo associated risk alleles across 5 super-populations and 26 ethnic populations belonging to the Phase 3 data of the 1000 Genomes Project (1KGP) in addition to 1029 individuals from the IndiGen Project [22]. Such comparative analysis may aid in prioritisation of risk alleles to analyse diseased cohorts belonging to other ethnicities. Additionally, allele frequency variation of prioritised SNPs belonging to vitiligo associated genes were also analyzed across populations in order to obtain a comprehensive understanding of disease prevalence as GWAS for vitiligo are scarce.

## Materials and methods

### Datasets

High coverage (30x) data belonging to 3202 samples (∼ 2 TB) from 1000 Genome Project comprising of 5 super-populations (African (AFR), Ad Mixed American (AMR), East Asian (EAS), European (EUR), and South Asian (SAS)) was used for the study [23]. Ad Mixed Americans were categorized into two sub-populations, ‘AMR1 (European derived, consisting of CLM and PUR populations) and AMR2 (Latino, comprising MXL and PEL populations) based on ancestry [24]. Allele frequency variations across populations pertaining to each super-population were also calculated using bcftools v1.9 [25]. To improve the representation of the genetic diversity from the Indian subcontinent, data representing genetic variation from 1029 healthy individuals across 27 states of the Indian subcontinent were extracted from the IndiGen database [22, 26]. Henceforth, these super-populations would be referred to as “7 super-populations” (Supplementary Table 01).

**Fig. 1:**
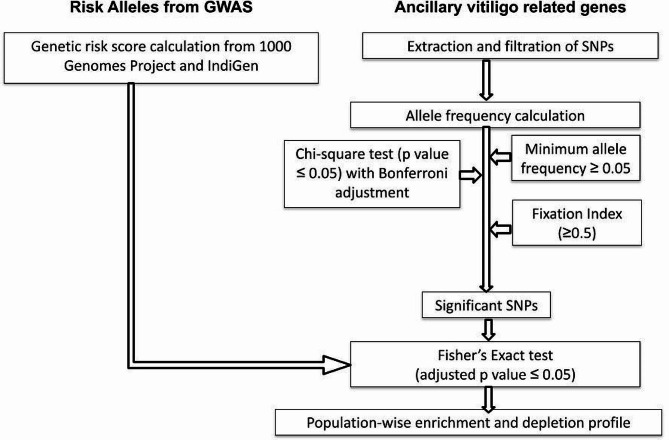
Flow-chart for population-wide estimation of enrichment and depletion of risk alleles and vitiligo associated genes

### Risk alleles from GWAS catalog

A total of 108 risk alleles associated with vitiligo from the NHGRI-EBI Catalog [21] of human GWAS were obtained. These risk alleles were obtained from eight studies belonging to European [13, 15, 27–31] and four studies belonging East Asian [32–35] ethnicities. Filtering based on odds ratio, risk allele frequency, redundant alleles and alleles absent in 1KGP, resulted in a total of 64 SNPs (62 and 2 SNPs belonging to European and East Asian GWAS respectively) belonging to 55 genes and were termed as ‘*risk alleles*’ (Supplementary Table 01) (Fig. 1).

### Compilation of vitiligo-associated genes

Databases such as Open Targets [36, 37] and VitVar [38, 39] were used to fetch 1079 vitiligo-associated genes and their corresponding 3,318,351 SNPs were derived from the 1KGP data.

### Genetic risk score calculation

Genetic risk score quantifies an individual’s genetic predisposition to a particular trait or disease based on their genetic information [11]. The following equation was used to calculate the genetic risk score for vitiligo, as described by [16].

$$\:Genetic\:Risk\:Score\:=\:\frac{\sum\:_{i=\:1\:}^{I}\:{X}_{i}}{2I}$$

where, I is the number of vitiligo SNPs and X_i_ is copies of risk alleles at i^th^ SNP.

### Bias analysis

Bias analysis was performed to eliminate concerns regarding genetic risk scores being biased towards the European and East Asian populations as the risk alleles used in this study have been derived from these populations. Hence, in order to understand the risk allele bias towards the said populations, three different sets of risk alleles (pertaining to the GWAS) were used for determining bias. Set1 includes 2 risk alleles from EAS and 8 random risk alleles from EUR; Set2 has 10 risk alleles from Set1 along with 5 risk alleles from EUR and Set3 has 10 random risk alleles from only EUR.

### Enrichment and depletion analysis

Fisher’s exact test was carried out to assess the effect of risk alleles in 7 super-populations and 26 sub-populations which were compared against the global 1KGP (referred to as *‘global normal’*). Fisher’s exact test was used to determine the enrichment and depletion of risk alleles in each population belonging to 1KGP and IndiGen as compared to the *global normal* risk allele score. This test is carried out by constructing a 2 × 2 contingency table where rows represent two different populations and columns represent presence and absence of alleles at given loci in two populations which are being compared. Log_10_ transformed adjusted *p*-value (≤ 0.05) was used for calculating depletion, whereas, to calculate enrichment, negative of the log_10_ transformed adjusted *p*-value (≤ 0.05) was used [17]. Seaborn and Matplotlib were used for generating heat maps [40, 41].

### Estimation of significant SNPs

Additional SNPs belonging to vitiligo-associated genes were prioritised using two-tailed Chi-square test and Bonferroni’s multiple adjustment values along with fixation index (F_st_≥0.5) and Minimum Allele Frequency (MAF ≥ 0.05) [42]. Chi-square test of significance quantifies the difference between observed and expected frequencies using the following formula:

$${{\rm{\chi }}^{\rm{2}}}{\rm{ = }}\,{\left( {{\rm{Obs}}\,{\rm{ - }}\,{\rm{Exp}}} \right)^{\rm{2}}}{\rm{/Exp}}$$

Fixation Index (F_st_) is a conventional metric used to detect allele frequency variation at population level [43]. F_st_ estimates the proportion of genetic variation at a specific locus between two populations. F_st_ is governed by common alleles (MAF) and size of each population.

### Annotation of risk alleles

Vannoportal, that provides in-depth annotation of variants (risk alleles) was used to analyse enriched/depleted alleles in order to ascertain if they are under positive selection [44]. The tests used for positive selection include χ^2^ test for Hardy-Weinberg equilibrium, Difference of Derived Allele Frequency, Fixation Index (Cockerham & Weir method), Integrated Haplotype score and Tajima’s D. Criterion of ≥ 3 tests indicating positive selection was used.

## Results

### SNPs identified from GWAS catalogue

A non-redundant set of 64 risk alleles (from EUR and EAS) belonging to 55 genes (Supplementary Table 01) were analysed. Vitiligo-associated genetic risk scores based on these risk alleles were calculated and their distribution across different super-populations were studied (Supplementary Fig. 01). As evident from this figure, the genetic risk scores (from GWAS) showed a similar range of distribution across the global population (normal samples). Hence, this suggests that the risk alleles obtained from the European and Han Chinese GWAS are suitable to investigate vitiligo prevalence in other population groups (Supplementary Fig. 01). Further in SAS, EUR, AMR1 and IndiGen the range of quartiles (Q1-Q3) possess similar values and EAS, AMR2 and AFR have marginally wider range.

**Fig. 2:**
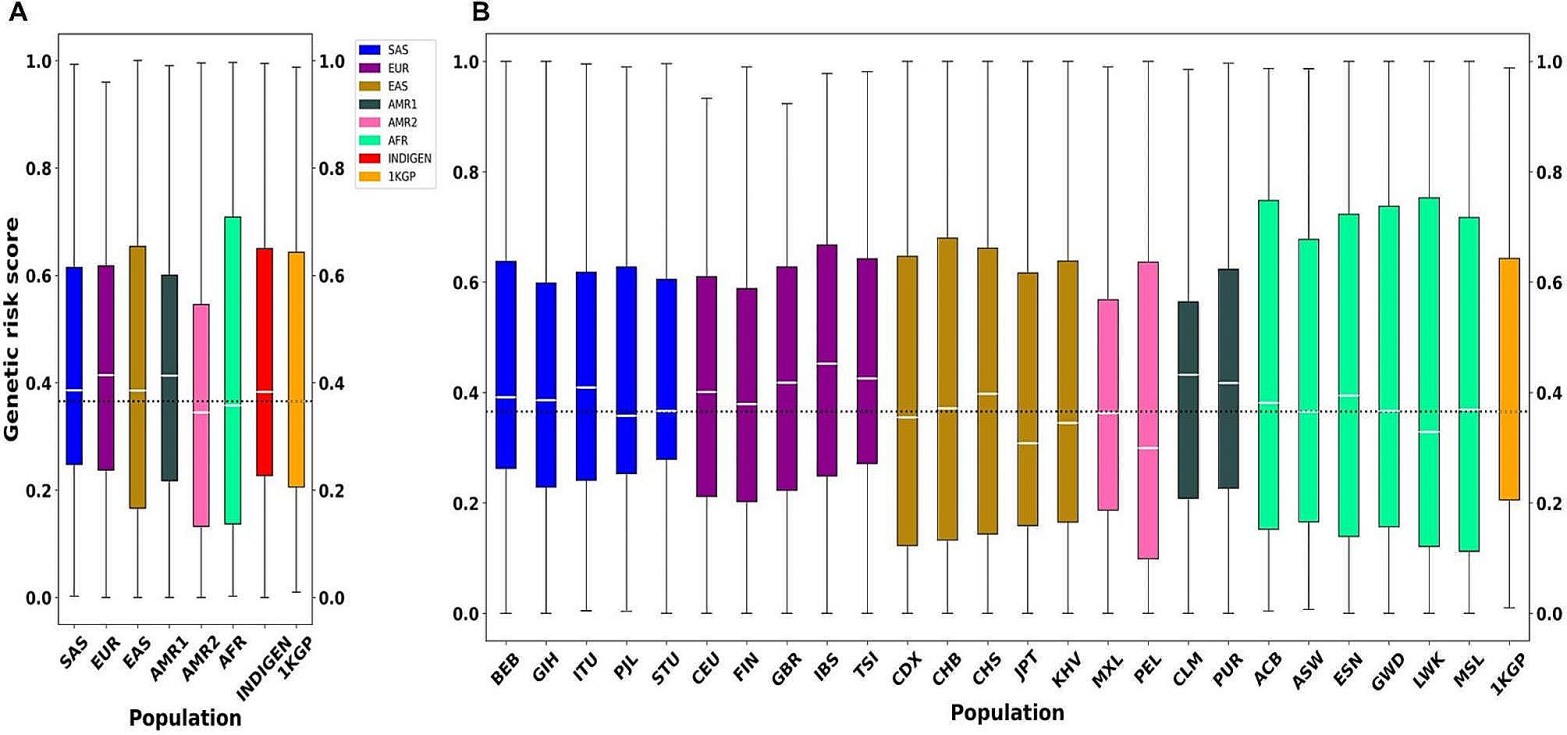
(**A**) Genetic Risk score obtained from GWAS for vitiligo across super-populations of 1000 Genomes and IndiGen projects. (**B**) Genetic Risk score obtained from GWAS for vitiligo across sub-populations of 1000 Genomes project. The dotted line represents the median of ‘*global normal’* risk score

### Genetic risk scores for SNPs related to vitiligo

The distribution of genetic risk scores for the six super-populations and 26 sub-populations belonging to 1KGP as well as IndiGen are shown in Fig. 2. Similar trends in the distribution of genetic risk scores were observed in SAS and IndiGen. The genetic risk score distribution of the AFR super-population tends towards upper quartile (Q2 and Q3) as compared to ‘*global normal’*, whereas, AMR2 tends towards lower quartile (Q1 and Q2). In case of SAS, EUR, EAS, AMR1 and IndiGen, genetic risk score was found to be in the range of ‘*global normal’* distribution (Fig. 2).

**Fig. 3:**
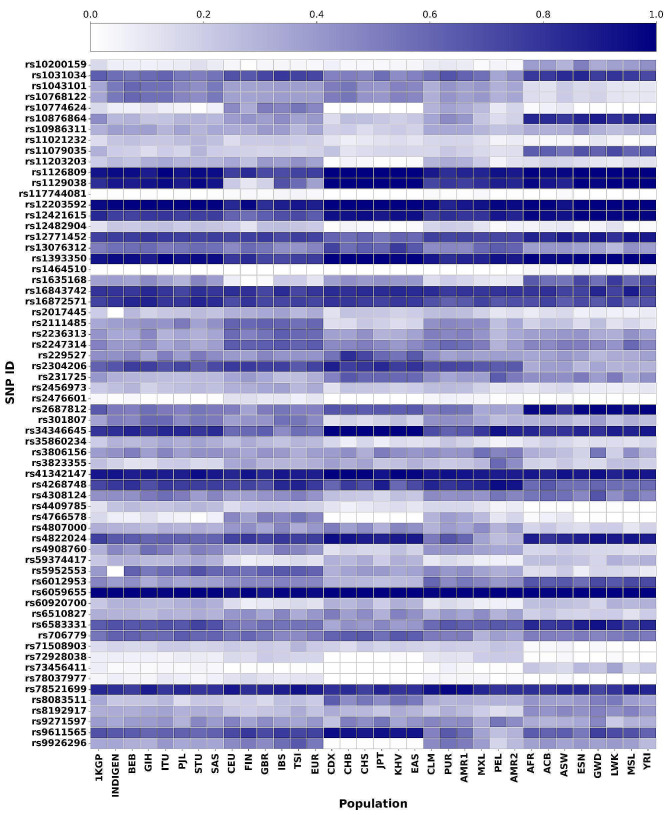
Heat map of genetic risk scores of vitiligo-associated risk alleles plotted for 1000 Genome and IndiGen projects

The genetic risk score is observed to follow a similar distribution for every sub-population pertaining to each super-population (Fig. 3). The score varies from 0.01 (rs117744081 belonging to gene *CPVL*) to 0.988 (rs6059655 belonging to gene *RALY*) (Fig. 3).

### Bias analysis

Comparison of genetic risk score distribution (based on all 64 risk alleles) of EUR with EAS revealed an enriched median of genetic risk score in EUR (Fig. 2).

**Fig. 4:**
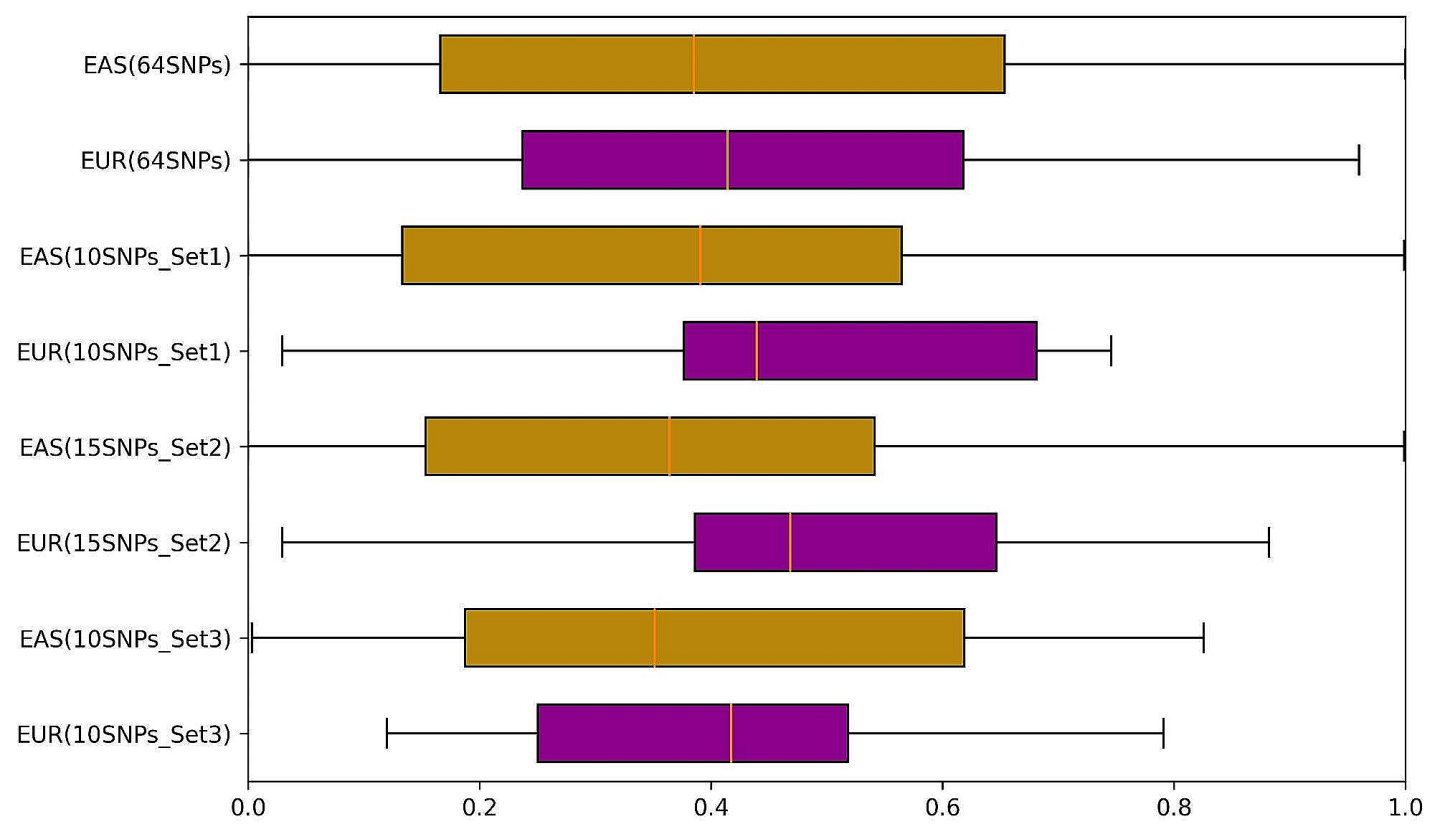
Distribution of genetic risk scores between EUR and EAS populations with different risk allele sets

In order to further validate this observation, three random sub-sets of risk alleles were sampled as described in the Methods section. The genetic risk score (median value) of these sub-sets for EAS and EUR along with the complete 64 risk alleles ranges between 0.35 and 0.39 and 0.41–0.46 respectively (Fig. 4; Table 1).

**Table 1:** Genetic risk score (median value) for EUR and EAS populations for three risk alleles sub-sets

Dataset	Number of Risk Alleles		Genetic risk score (median value)	
	EUR	EAS	EUR	EAS
Set1	10	10	0.42	0.35
Set2	15	15	0.47	0.36
Set3	10	10	0.44	0.39
All risk alleles	64	64	0.41	0.39

### Enriched and depleted risk alleles across super-populations

**Fig. 5:**
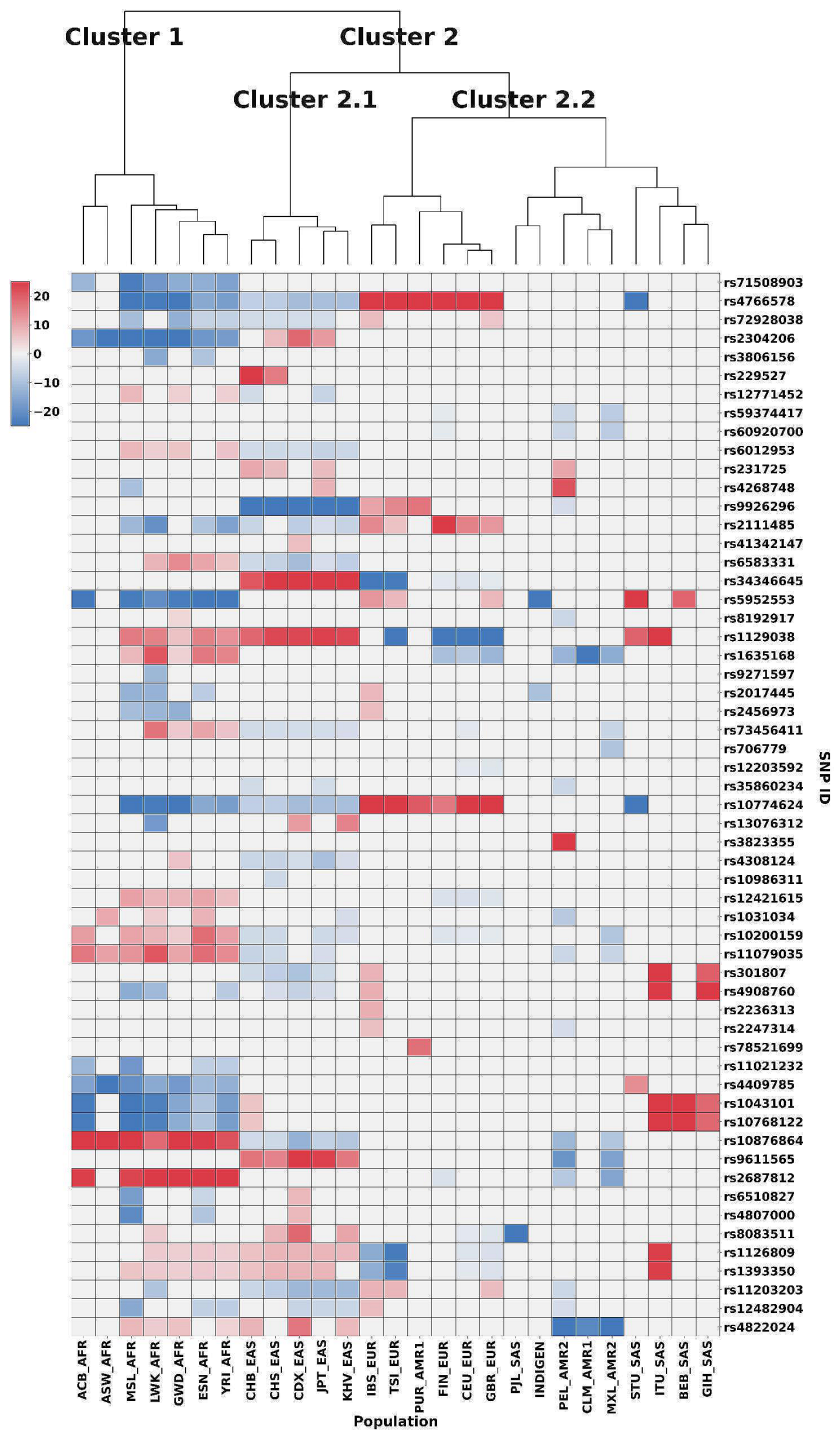
Heatmap of vitiligo-associated risk alleles from 1000 Genomes and IndiGen projects. Red and blue colours represent enriched and depleted effect alleles respectively

Based on enrichment and depletion values of the risk alleles, two distinct clusters were observed with AFR being the outermost branch (cluster 1) (Fig. 5). In cluster 2, all other populations group together, in which EAS forms a separate branch (sub-cluster 2.1). EUR, AMR1, AMR2, SAS along with IndiGen cluster together (sub-cluster 2.2). AMR1 was found to group with both EUR and SAS, whereas, AMR2 clustered along with the South Asian populations (Fig. 5). Of the 64 risk alleles, 57 are either enriched or depleted or satisfy both conditions in at least one population, whereas the remaining seven risk alleles were found to have no significant change and hence were excluded from the heatmap (Fig. 5).

The risk alleles which contribute to the clustering include, rs10876864 (TF binding site variant of *IKZF4*, *SUOX* and involved in disease like asthma, allergic diseases, squamous cell carcinoma, vitiligo), rs2687812 (intron variant of *TG* and involved in vitiligo) and rs34346645 (intron variant of *FOXP1* involved in vitiligo), rs9611565 (intron variant of *TEF* involved in vitiligo) which are found to be highly enriched only in AFR and EAS populations respectively. Similarly, rs71508903 (intron variant of *ARID5B* involved in rheumatoid arthritis, hypothyroidism and vitiligo), rs2304206 (5 prime UTR variant of *BCL2L12*, *IRF3* involved in vitiligo), rs4409785 (intron variant of *LNCRNA-IUR*, *FAM76B* and involved in rheumatoid arthritis, multiple sclerosis, autoimmune thyroid disease, basal cell carcinoma, vitiligo), rs1043101, rs10768122 (3 prime UTR variant of *SLC1A2* involved in vitiligo) are found to be depleted in AFR. rs9926296 (intron variant of *FANCA* and involved in vitiligo) is found to be depleted only in EAS.

Risk alleles rs1043101 and rs10768122 are found to be highly enriched in SAS (BEB, GIH and ITU) while depleted in AFR. Risk alleles rs301807 and rs4908760 (intron variants of *RERE*) were observed to be highly enriched in SAS (GIH and ITU) and depleted in EAS. Risk alleles rs1393350 (intron variant of *TYR*) and rs1126809 (missense variant of *TYR*) are observed to be highly enriched in ITU (SAS) and moderately enriched in AFR and EAS while being depleted in EUR. rs1129038 (3 prime UTR variant from *HERC2*) is found to be enriched in SAS (STU, ITU) along with all sub-populations of EAS and AFR, however it was found to be depleted in EUR. rs4409785 (intron variant of *FAM76B*) is observed to be enriched only in STU (SAS) and depleted in AFR. rs5952553 (intergenic variant *GAGE1* - *VDAC1P2*) is found to be enriched in SAS (STU, BEB) and EUR while it was depleted in IndiGen and AFR.

Conversely, risk alleles rs4766578 and rs10774624 (intron variants of *ATXN2*) were found to be depleted in STU (SAS), EAS and AFR while these were enriched in EUR. Risk allele rs10774624 was found to be under positive selection in EUR (Supplementary Table 01). Risk allele rs2017445 (intron variant of *IKZF4*) is depleted in IndiGen, AFR and found to be enriched in IBS (EUR). rs8083511 (intron variant of *TNFRSF11A*) is depleted in PJL (SAS) and enriched in EAS and AFR.

Enrichment/depletion patterns of risk alleles belonging to cluster 2.2 (EUR, SAS, AMR1, AMR2 and IndiGen) have been analysed in detail as higher prevalence of vitiligo has been reported earlier in South Asian populations [2] (Table 2). The details of the enrichment/depletion of risk alleles in SAS are given below.

**Table 2:** Enrichment and depletion profiles of genetic risk scores across populations from IndiGen and 1000 Genomes projects in Vitiligo-associated risk alleles. Association of risk alleles with other diseases is detailed

Population	IndiGen	South Asian					European					Admixed Americans				Role of vitiligo-associated risk alleles in other diseases (PubMed ID)
												AMR1		AMR2		
ID	IndiGen	BEB	GIH	ITU	PJL	STU	CEU	FIN	GBR	IBS	TSI	CLM	PUR	MXL	PEL	
rs1043101	↔	↑	↑	↑	↔	↔	↔	↔	↔	↔	↔	↔	↔	↔	↔	NA
rs10768122	↔	↑	↑	↑	↔	↔	↔	↔	↔	↔	↔	↔	↔	↔	↔	NA
rs10774624	↔	↔	↔	↔	↔	↓	↑	↑	↑	↑	↑	↔	↑	↔	↔	Heart failure (36376295), Intraocular pressure (29785010), Peripheral artery disease in non diabetes (34601942), Preeclampsia (33239696, 37248299), Rheumatoid arthritis (24390342), Systolic blood pressure (30578418), Gamma interferon levels (27989323)
rs1126809	↔	↔	↔	↑	↔	↔	↓	↔	↓	↓	↓	↔	↔	↔	↔	Aging traits (32678081), Cutaneous melanoma or hair colour (32341527), Eye color (33692100), Nevus count or cutaneous melanoma (32341527), Sunburns (23548203), Tanning (23548203)
rs1129038	↔	↔	↔	↑	↔	↑	↓	↓	↓	↔	↓	↔	↔	↔	↔	Corneal astigmatism (30306274), Refractive astigmatism (30306274), Uveal melanoma (34424336)
rs1393350	↔	↔	↔	↑	↔	↔	↓	↔	↓	↓	↓	↔	↔	↔	↔	NA
rs2017445	↓	↔	↔	↔	↔	↔	↔	↔	↔	↑	↔	↔	↔	↔	↔	NA
rs301807	↔	↔	↑	↑	↔	↔	↔	↔	↔	↑	↔	↔	↔	↔	↔	Depression (29942085), Eosinophil counts (35935937)
rs4409785	↔	↔	↔	↔	↔	↑	↔	↔	↔	↔	↔	↔	↔	↔	↔	Autoimmune thyroid disease (32581359, 22922229), Basal cell carcinoma (36496446), Graves’ disease (34594039, 22922229), Hypothyroidism (34594039), Lymphocyte side scatter (37596262), Multiple sclerosis (31604244), Myasthenia gravis (35074870), Rheumatoid arthritis (24390342, 36333501), Eosinophil counts (32888493, 32888494), Thyroid preparations (34594039, 31015401), Sex hormone-binding globulin levels (33462484, 34321204)
rs4766578	↔	↔	↔	↔	↔	↓	↑	↑	↑	↑	↑	↔	↑	↔	↔	Apolipoprotein B levels (35213538), Arthritis (33106285), Cholesterol level (36764567, 35213538), Heart failure (31919418), Right ventricular stroke volume (37126556), Sjögren’s syndrome (24097067), Smoking (33082346, 32231276), Total omega-6 fatty acid levels, Linoleic acid levels (35692035)
rs4908760	↔	↔	↑	↑	↔	↔	↔	↔	↔	↑	↔	↔	↔	↔	↔	NA
rs5952553	↓	↑	↔	↔	↔	↑	↔	↔	↑	↑	↑	↔	↔	↔	↔	NA
rs8083511	↔	↔	↔	↔	↓	↔	↓	↔	↓	↔	↔	↔	↔	↔	↔	NA

↔ indicates no significant change; ↑ indicates enriched; ↓ indicates depleted

#### Risk alleles found to be depleted in SAS

Risk allele rs4766578 (intron variant of *ATXN2*) is found to be depleted in SAS, EAS and AFR and enriched in EUR and AMR1. This risk allele is found to be under positive selection in SAS, AMR1 and EUR (Supplementary Table 01). Apart from being associated with vitiligo susceptibility, this SNP is also found to be a risk allele for diastolic blood pressure and coronary artery disease in Europeans [6, 28, 45, 46]. Recently this SNP has been associated with a role in positive selection by regulating *ALDH2* gene expression that protects cells from acetaldehyde toxicity in the European population [47]. rs10774624 is found to be depleted in SAS, EAS and AFR and enriched in EUR and AMR1. This risk allele is found to be associated with rheumatoid arthritis in Pakistanis and in severe COVID-19 patients from the UK Biobank cohort [48, 49].

#### Risk alleles found to be enriched in SAS

Risk alleles rs1043101, rs10768122 (3 prime UTR variants of *SLC1A2*) and rs4409785 (intron variant of *FAM76B*) were found to be enriched in SAS and depleted in AFR. These SNPs have been associated with other disorders apart from vitiligo susceptibility. Risk allele rs1043101, is found to be associated with bipolar disorder and schizophrenia in European populations [50]. rs4409785 has been found to be associated with Graves’ disease in Europeans, multiple autoimmune diseases in European Americans, rheumatoid arthritis in Pakistanis and myasthenia gravis in cohort of UK Biobank [48, 51–53].

rs1129038 (3 prime UTR variants of *HERC2*) is found to be enriched in SAS, EAS, AFR and depleted in EUR. This risk allele is predicted to be under positive selection in EUR (Supplementary Table 01). It is found to be associated with eye colour in Europeans, skin pigmentation in Brazilians, susceptibility to myopia in UK Biobank, uveal melanoma risk in Americans [54–60]. rs4908760 (intron variant of *RERE*) is found to be enriched in SAS whereas it is depleted in EAS, AFR and has association with smoking behaviour-related traits [61]. rs301807 (intron variant of *RERE*) is found to be enriched in SAS and depleted in EAS. rs5952553 (intergenic variant of *GAGE1* - *VDAC1P2*) is found to be enriched in SAS and EUR while being depleted in AFR. A non-synonymous variant rs1126809 (*TYR*) is found to be highly enriched in ITU (SAS) and moderately enriched in AFR and EAS while being depleted in EUR. This SNP has correlation with longitude, latitude, sunshine hours in the Chinese population, cancer among the South-East Asians, brown eye colour in Europeans and increased risk of melanoma in south Brazilians [57, 62–64]. rs1393350 (*TYR*) is found to be highly enriched in ITU (SAS) and moderately enriched in AFR and EAS while depleted in EUR. It is found to be associated with eye and hair colour in the Slovenians, skin colour in the Europeans and eye colour in the Pakistanis [65–69].

Similarly, the details of enriched and depleted risk alleles in EUR, EAS, AMR1, AMR2 and AFR are provided in Supplementary document S1.

#### Risk alleles under positive selection

Apart from the above-mentioned risk alleles that are found to be enriched/depleted in SAS and under positive selection, few more risk alleles are also observed to meet the selection criterion. rs1635168 (intron variant of *HERC2*) in EUR, rs9926296 (intron variant of *FANCA*) in EAS, rs2304206 (5 prime UTR variant of *IRF3*) and rs2111485 (intron variant of *IFIH1*) in AFR are depleted and found to be under positive selection (Supplementary Table 01).

rs2111485 in EUR and rs10200159 (non-coding transcript exon variant of *PPP4R3B*), rs10876864 (TF binding site variant of *IKZF4*), rs2687812 (intron variant of *TG*), rs12771452 (intron variant of *CASP7*) and rs11079035 (intron variant of *RAB5C*) in AFR are enriched and found to be under positive selection (Supplementary Table 01).

Overall, the highest number of enriched risk alleles are observed in AFR super-population followed by EAS and EUR (Table 3). It is interesting to note that none of the risk alleles are enriched in IndiGen. The highest percentage of depleted risk alleles are observed in EAS followed by AMR2 and AFR.

**Table 3:** Percentage of depleted and enriched risk alleles in different super-populations

	INDIGEN	SAS	EUR	EAS	AMR1	AMR2	AFR
Depleted	3.13	0.94	10.63	23.75	1.56	20.31	18.30
Enriched	0.00	5.31	10.00	14.38	3.13	2.34	16.74

### Analysis of ancillary vitiligo-associated genes

A comprehensive list of 1079 genes associated with vitiligo, were compiled from two databases as mentioned in the Methods section. These genes include 3,318,351 SNPs from the 1KGP data with GRCh38 as reference. Among these SNPs, 3,195,407 were found to be biallelic. Of these, 143,562 SNPs satisfied the MAF ≥ 0.05 in all populations. To understand the preponderance of vitiligo in SAS, 197,543 SNPs with MAF ≥ 0.05 were further extracted (Supplementary Table 02). Significant allele frequency variation was observed in 117 SNPs belonging to 44 genes (Supplementary Fig. 02). MAF ≥ 0.05 ensured that the selected SNPs were relatively common in the population with Fst ≥ 0.5. To account for multiple testing, Bonferroni’s correction, a stringent method for controlling the family-wise error rate was used, which further validated the significance of prioritised SNPs. It is envisaged that these SNPs represent a subset of genetic variants that warrant special attention, as they are likely to play a critical role in vitiligo. Enrichment and depletion analysis of these SNPs was performed as described previously in the Methods section for which allele frequencies were retrieved from 1KGP (Supplementary Fig. 03). Both the distribution patterns (allele frequency distribution and enrichment/depletion of alleles) reveal distinct population-specific clustering (Supplementary Figs. 02 and 03).

### Functional annotation of the obtained SNPs

Of the 117 SNPs, only one SNP is non-synonymous, nine are downstream, 11 are upstream and 96 are intron variants (Supplementary Table 01). All 117 SNPs are either enriched or depleted for one or more populations. Missense variant rs16891982 belonging to *SLC45A2* gene is enriched in EUR and AMR1 super-populations and depleted in all others. This SNP has been reported to be associated with skin pigmentation disorder [70]. Enrichment analysis shows that the maximum enriched SNPs are in EAS followed by AFR, AMR2, EUR, SAS, AMR1 and IndiGen (Supplementary Fig. 03).

## Discussion

There exist varying reports pertaining to the preponderance of vitiligo across populations [71, 72]. In this study, we compared the allele frequencies of vitiligo-associated risk alleles across different populations to gain insight into the role of genetic variation in estimating disease risk. The enrichment and depletion patterns of 64 vitiligo-associated risk alleles quantifies genetic variations and its prevalence in populations (from 1KGP and IndiGen) as compared to ‘*global normal*’. Several studies have reported variants identified through GWAS to be multi-ethnically reproducible [17, 73]. GWAS pertaining to vitiligo are majorly confined to European populations [6]. The calculated genetic risk scores in this study obtained across super-populations of 1KGP reveal similar distribution, thereby suggesting the relevance of these risk alleles to be analysed across ethnicities (Supplementary Fig. 01). Further, bias analysis unveiled the effect of allele frequencies to be a significant factor that determines the outcome of genetic risk scores and not the mere occurrence of vitiligo-associated risk alleles.

Population-specific genetic risk scores of few alleles (which are also associated with other autoimmune diseases (Table 2)) were observed to be higher in normal populations when compared against the ‘*global normal*’. For instance, rs4766578 belonging to gene *ATXN2* (contributing towards the pathogenesis of vitiligo, cardiovascular diseases and involved in haematological parameters governing platelet counts and volume) and rs10774624 (involved in rheumatoid arthritis, preeclampsia, heart diseases) was found to be highly enriched in EUR and AMR1 populations and depleted in AFR and EAS thereby indicating the frequent occurrence of these alleles in the populations belonging to European ancestry [6, 24, 45, 46, 74, 75]. Both these risk alleles are also found to be under positive selection (Supplementary Table 01). Conventionally in complex diseases minor alleles are associated with disease risk [76]. Many of the risk alleles chosen in this study have reported odds-ratio of ∼[1-1.1] and hence their allele frequencies are close to normal population (Supplementary Table 01).

The higher proportion of enriched alleles in AFR obtained in this study corroborates with meta-analysis of vitiligo prevalence [77]. Lower percentage of enriched risk alleles in SAS and IndiGen observed in our study does not substantiate reports of higher prevalence of vitiligo in this region [2]. Further, enrichment and depletion analysis revealed AFR and EAS to form separate clusters indicating their ethnicity-specific genetic variation. This clustering can be explained by the ‘Out of Africa’ hypothesis, which proposes East Africa being the cradle for the origin of modern humans [78]. Earlier studies have shown the African populations to be genetically most diverse [79]. Genetic diversity studies predicted the origin of humans along with their migration routes to subsequent human expansion from East Africa [80]. The heterozygosities and the observed patterns of genetic diversities existing in the global populations are explained due to this expansion originating from Africa. With the increasing geographic distance, a decrease in genetic similarity is observed between the populations due to geographic isolation (measured as *F*_*ST*_), genetic drift and natural selection [81]. The ‘trellis model’ based on genetic distances existing among human populations proposes Africans and Asians to be genetically distant, which is also reiterated in our study, wherein AFR and EAS are found to cluster independently (Fig. 5) [78]. These prioritized risk alleles may be investigated further while performing population-specific vitiligo related GWA studies.

Clustering of AMR1 with EUR explains its European ancestry [74]. The observed grouping of CLM (belonging to AMR1 - Admixed Americans from the Colombian/Peruvian/Mexican group) with AMR2 supports the reports suggesting CLM to be closely related to the Peruvian (PEL) population [74]. IndiGen and PJL (belonging to SAS) cluster together and their enrichment and depletion profiles were found to be similar to the ‘*global normal*’. SAS sub-populations (except for PJL) are observed to cluster together and are in close proximity to AMR2 along with CLM (belonging to AMR1). This clustering may be due to a large number of risk alleles having similar scores as ‘*global normal*’ (Fig. 5). Overall, the clustering of SAS, IndiGen with EUR can be attributed to majority of the gene pool belonging to the ancient north Indian ancestry which is known to be genetically close to Europeans [75, 82].

It should be mentioned that as vitiligo is a polygenic disorder, enrichment/depletion of individual risk alleles may have limited scope to explain aetiology. Estimation of polygenic risk score may provide a better insight into disease susceptibility. Additionally, the pleiotropic effect of risk alleles may also play a role in their selection especially in auto immune diseases [83].

As limited GWA studies are available for populations belonging to other ethnicities apart from EAS and EUR, additional SNPs belonging to vitiligo-associated disorder were also studied. Allele frequency distribution pattern along with enrichment and depletion patterns revealed distinct population-specific clustering (Supplementary Figs. 02 and 03). Individuals from the IndiGen cohort are observed to cluster along with the South Asian population from 1KGP indicating similar trends in allele frequencies (Supplementary Figs. 02 and 03). rs2279238 and rs11039155 belonging to the *LXR-α* gene involved in regulation of metalloproteinase 2 and 9 (melanocyte precursors) are associated with vitiligo risk in the North Indian population [84]. The allele frequency distribution of rs2279238 and rs11039155 was found to be 0.2 and 0.1 respectively in populations belonging to the SAS and IndiGen cohort, which is in agreement to earlier reports from India (Supplementary Table 02) [84]. This elucidates the importance of identification and study of other functional SNPs related to genes involved in vitiligo. Hence, such large-scale comparisons of allele frequencies across different population groups can provide additional markers for population-specific GWA studies.

It needs to be noted that interpretation of the role of GWAS risk alleles across population may lead to over/under-estimation of disease risk and hence adequate caution has to be taken [85]. Overall, the prioritized risk alleles and additional SNPs identified in this study can play a role in designing precision public health initiatives for tackling vitiligo. Such population-specific variants may help in screening for vitiligo prevalence.

## Conclusion

The comprehensive analysis of vitiligo-associated risk alleles for enrichment and depletion across diverse populations reveals intriguing patterns. Notably, many variants are observed to be differentially enriched/depleted in various populations which is indicative of intricate inter-population variations. The risk alleles were obtained primarily from the EUR population, highlighting the need for expanded investigations across varied ethnicities to gain comprehensive insights. These risk alleles, linked to vitiligo, are not only associated with the disease but are also implicated in other autoimmune conditions, emphasising their role in a broader disease spectrum. Additional sets of vitiligo-associated SNPs identified based on allele frequency variation can complement GWAS. These findings collectively emphasise the importance of considering genetic diversity and population-specific factors when evaluating disease risk.

### Electronic supplementary material

Below is the link to the electronic supplementary material.

Supplementary document S1: Enriched and depleted risk alleles in EAS, EUR, AMR1, AMR2 and AFR super-populations

Supplementary Figure 01: Comparison of vitiligo associated genetic risk scores from GWAS across super-populations reported in 1000 Genome and IndiGenomes Project

Supplementary Figure 02: Distribution of vitiligo associated SNP frequency from prominent databases and literature across super-populations / populations reported in 1000 Genome and IndiGenomes project

Supplementary Figure 03: Enriched and depleted pattern of significant variation in 117 SNPs of vitiligo in across populations reported in 1000 Genome and IndiGenomes project

Supplementary Table 01: Details of vitiligo-associated risk alleles, computed risk allele scores in 1KGP and IndiGen populations and their sites under positive selection as predicted by VannoPortal

Supplementary Table 02: SNPs belonging to additional vitiligo-associated genes in 1KGP super-populations (filtered based on MAF ≥ 0.05 in South Asian super-population) and annotation of significant SNPs

## Data Availability

Data analyzed in this study is provided as additional Supplementary material.

## Abbreviations

SNPs: Single nucleotide polymorphisms
MAF: Minimum Allele Frequency
GWAS: Genome wide association studies
1KGP: 1000 Genomes Project
NHGRI-EBI: National Human Genome Research Institute-European Bioinformatics Institute
EAS: East Asian Ancestry
EUR: European Ancestry
AFR: African Ancestry
AMR: Admixed American Ancestry
SAS: South Asian Ancestry
CHB: Han Chinese in Beijing, China
JPT: Japanese in Tokyo, Japan
CHS: Han Chinese South
CDX: Dai in Xishuangbanna, China
KHV: Kinh in Ho Chi Minh City, Vietnam
CEU: Utah residents (CEPH) with Northern and Western European ancestry
TSI: Toscani in Italia
GBR: British in England and Scotland
FIN: Finnish in Finland
IBS: Iberian populations in Spain
YRI: Yoruba in Ibadan, Nigeria
LWK: Luhya in Webuye, Kenya
GWD: Gambian in Western Division, The Gambia
MSL: Mende in Sierra Leone
ESN: Esan in Nigeria
ASW: African Ancestry in Southwest US
ACB: African Caribbean in Barbados
MXL: Mexican Ancestry in Los Angeles, California
PUR: Puerto Rican in Puerto Rico
CLM: Colombian in Medellin, Colombia
PEL: Peruvian in Lima, Peru
GIH: Gujarati Indian in Houston, TX
PJL: Punjabi in Lahore, Pakistan
BEB: Bengali in Bangladesh
STU: Sri Lankan Tamil in the UK
ITU: Indian Telugu in the UK
