# Supplementary material for: Estimation of genetic variation in vitiligo associated genes: Population genomics perspective
Source: BMC Genom Data. 2024 Jul 26;25:72. doi: 10.1186/s12863-024-01254-6 (PMC11282599; doi:10.1186/s12863-024-01254-6)
Supplement: Supplementary file 1 — Supplementary document S1: Enriched and depleted risk alleles in EAS, EUR, AMR1, AMR2 and AFR super-populations [file 12863_2024_1254_MOESM1_ESM.docx]

**Supplementary document S1** : Enriched and depleted risk alleles in EAS, EUR, AMR1, AMR2 and AFR super-populations

***Depleted in EUR:***

rs8083511 andrs34346645 are depleted in EUR and enriched in EAS. rs8083511 is associated with the distal radius cross-sectional area in the forearm in men in the European population [[1]](https://www.zotero.org/google-docs/?9aKm9j) rs1635168 is depleted in EUR, AMR1, AMR2 and enriched in AFR. It is found to be associated with eye pigmentation in the European population [[2]](https://www.zotero.org/google-docs/?2d0jMz) and iris colour in the Netherlands population [[3]](https://www.zotero.org/google-docs/?RdRXL6) and also found to be associated with hair color in the European population [[4]](https://www.zotero.org/google-docs/?MAf2UI)**.**

rs10200159 and rs1393350 are depleted in EUR, EAS and enriched in AFR. rs1393350 haplotype analysis showed a strong association in vitiligo patients [[5]](https://www.zotero.org/google-docs/?kbGvVG), in Slovenian population found to be major genetic predictors of eye color [[6,7]](https://www.zotero.org/google-docs/?nVDxqa), eye and hair color in the Slovenian population [[8]](https://www.zotero.org/google-docs/?ziLI6f), identified as risk factors for actinic keratosis for skin color in the European population [[9]](https://www.zotero.org/google-docs/?E4rzvo), with significant association with eye colour in the Pakistani population [[10]](https://www.zotero.org/google-docs/?uQ4vRN).

A non synonymous variant rs1126809 is depleted in EUR and enriched in EAS and AFR. This SNP has correlation with longitude, latitude, sunshine hours in Chinese population [[11]](https://www.zotero.org/google-docs/?gHspky), also found to be a cancer pathogenic variant with increase risks of cancer among the Orang Asli and Malays [[12]](https://www.zotero.org/google-docs/?83Fkh1), is associated with brown eye colour [[13]](https://www.zotero.org/google-docs/?cWmNOK), observed increased risk of melanoma in southern Brazil [[14]](https://www.zotero.org/google-docs/?Pmcl94). rs12421615 depleted in EUR and enriched in AFR. rs12203592 is depleted in EUR only and was not found to be enriched in any population. This SNP plays a crucial role in the modulation of melanoma resulting primary tumour in European population [[15–17]](https://www.zotero.org/google-docs/?kSdmWk), associated with susceptibility to skin cancer and haematological malignancies, increased risk of skin cancer among Caucasians [[18]](https://www.zotero.org/google-docs/?eZYFWe) and in a Spanish population is found to be with melanoma susceptibility [[19]](https://www.zotero.org/google-docs/?wDAshA).

***Enriched in EUR:***

rs10774624, rs4766578, rs5952553 enriched in EUR and have been discussed previously. Risk alleles of rs10774624, rs4766578 and rs5952553 SNPs are enriched over the global 1KGP population. rs2111485 and rs72928038 are enriched in EUR whereas these are depleted in EAS and AFR. G allele of rs2111485 is found to be protective for vitiligo development in the Turkish population [[20]](https://www.zotero.org/google-docs/?G4oTEt), it is also associated in the Chinese Han population for spontaneous clearance of hepatitis B virus [[21]](https://www.zotero.org/google-docs/?XZgdsj). rs2111485 [[22]](https://www.zotero.org/google-docs/?09Ju4u) and rs72928038 [[23]](https://www.zotero.org/google-docs/?YohOCi) are associated with type 1 diabetes risk in the Polish population. rs9926296 is enriched in EUR and AMR1 whereas it is depleted in EAS. This SNP is associated with melanoma risk in the US population [[24]](https://www.zotero.org/google-docs/?CZ3qgD).

***Depleted in EAS:***

rs10200159, rs10774624, rs2111485, rs301807,rs4766578, rs4908760, rs72928038, rs9926296 are depleted in EAS population and are discussed earlier. Rs10876864, rs11079035, rs1031034 are depleted in EAS and AMR2 and enriched in AFR. rs10876864 is associated with susceptibility for vitiligo in the Chinese Han population [[25]](https://www.zotero.org/google-docs/?bnFm0t) and is also associated with type 1 diabetes in the Spanish population [[26]](https://www.zotero.org/google-docs/?3tWyXi). Risk alleles of rs11203203, rs35860234, rs4308124 SNPs are found to be depleted in EAS only. rs12482904 is depleted in both EAS and AFR. rs12771452, rs73456411, rs6012953 and rs6583331 are depleted in EAS and enriched in AFR.

rs11079035, rs1031034, rs35860234, rs4308124, rs12482904, rs12771452, rs73456411, rs6012953, rs6583331 no literature.

rs11203203 is found to be predictor of islet autoimmunity and type 1 diabetes in non-Hispanic white children [[27,28]](https://www.zotero.org/google-docs/?cyje1R), however in a Chinese population, there was not significant association with rheumatoid arthritis susceptibility and autoantibody profiles [[29]](https://www.zotero.org/google-docs/?8CoBO2). In another study [[30]](https://www.zotero.org/google-docs/?wdmIh8) rs11203203 with interaction rs2476601 are associated with Type 1 Diabetes.

***Enriched in EAS:***

rs1126809, rs1129038, rs1393350, rs34346645 and rs8083511 are enriched in EAS, and are discussed previously. rs229527 and rs231725 are enriched in EAS only. rs2304206 and rs4807000 are enriched in EAS and depleted in AFR. rs4822024 is enriched in EAS, AFR and depleted in AMR1, AMR2. rs9611565 is enriched in EAS and depleted in AMR2. rs4268748 and rs2017445 are enriched in EAS and AMR2 while they are depleted in AFR. rs229527 is found to have significant associations with risk of Graves disease in the Han Chinese population [[31]](https://www.zotero.org/google-docs/?uDdEdu).

rs231725 shows as a risk factor of primary biliary cholangitis in the Chinese population [[32,33]](https://www.zotero.org/google-docs/?A6gR61) and in the Japanese population [[34,35]](https://www.zotero.org/google-docs/?FNT5YL). while in another study it has negative association with primary biliary cholangitis [[36]](https://www.zotero.org/google-docs/?dnxFuf). This SNP is also associated with chronic obstructive pulmonary disease [[37]](https://www.zotero.org/google-docs/?DK9zxa), Allergic rhinitis with asthma [[38]](https://www.zotero.org/google-docs/?vfLgcS) in the Chinese population. rs2304206 is not associated with hepatitis B virus infection in Chinese patients [[39]](https://www.zotero.org/google-docs/?xGyu72), in another study risk allele rs2304206 associated with increased susceptibility to systemic lupus erythematosus in Mexican mestizo population [[40]](https://www.zotero.org/google-docs/?lCowxX).

***Depleted in AMR1:***

rs1635168 and rs4822024 are depleted in AMR1 and discussed earlier.

***Enriched in AMR1:***

rs10774624, rs476657 and rs9926296 are enriched in AMR1 and discussed in this section in an earlier paragraph. while only one risk allele of rs78521699 SNP enriched in AMR1.

***Depleted in AMR2:***

rs10876864, rs11079035, rs1635168, rs4822024 and rs9611565 are depleted in AMR2 and discussed earlier. rs2687812 is depleted in AMR2 and enriched in AFR. rs59374417 and rs60920700 is depleted in AMR2 only

***Enriched in AMR2****:*

rs4268748 is enriched in AMR2 and discussed earlier. rs2456973 is enriched in AMR2 while it is depleted in AFR. rs3823355 is enriched in only AMR2. Risk allele of rs2456973 has association with Type 1 Diabetes Risk In Caucasian population [[41]](https://www.zotero.org/google-docs/?ezkHR7).

***Depleted in AFR:***

rs1043101, rs10768122, rs10774624, rs12482904, rs2017445, rs2111485, rs2304206, rs2456973, rs4409785, rs4766578, rs4908760, rs5952553, rs72928038 and rs4268748 are depleted in AFR and discussed earlier. rs11021232, rs3806156 and rs71508903 are depleted only in AFR.

***Enriched in AFR:***

rs10200159, rs1031034, rs10876864, rs11079035, rs1126809, rs1129038, rs12421615, rs12771452, rs1393350, rs1635168, rs2687812, rs4822024, rs6012953, rs6583331 and rs73456411 are enriched in AFR and discussed previously.

**List of abbreviations**

***EAS:*** East Asian Ancestry

***EUR:*** European Ancestry

***AFR:*** African Ancestry

***AMR:*** Admixed American Ancestry

***SAS:*** South Asian Ancestry

**References:**

[1. Roshandel D, Holliday KL, Pye SR, Ward KA, Boonen S, Vanderschueren D, Borghs H, Huhtaniemi IT, Adams JE, Bartfai G, Casanueva FF, Finn JD, Forti G, Giwercman A, Han TS, Kula K, Lean ME, Pendleton N, Punab M, Silman AJ, Wu FC, Thomson W, ONeill TW; EMAS Study Group. Influence of polymorphisms in the RANKL/RANK/OPG signaling pathway on volumetric bone mineral density and bone geometry at the forearm in men. Calcif Tissue Int. 2011 Dec;89(6):446-55. doi: 10.1007/s00223-011-9532-y.](https://www.zotero.org/google-docs/?ONeNiz)

[2. Candille SI, Absher DM, Beleza S, Bauchet M, McEvoy B, Garrison NA, Li JZ, Myers RM, Barsh GS, Tang H, Shriver MD. Genome-wide association studies of quantitatively measured skin, hair, and eye pigmentation in four European populations. PLoS One. 2012;7(10):e48294. doi: 10.1371/journal.pone.0048294.](https://www.zotero.org/google-docs/?ONeNiz)

[3. Kayser M, Liu F, Janssens AC, Rivadeneira F, Lao O, van Duijn K, Vermeulen M, Arp P, Jhamai MM, van Ijcken WF, den Dunnen JT, Heath S, Zelenika D, Despriet DD, Klaver CC, Vingerling JR, de Jong PT, Hofman A, Aulchenko YS, Uitterlinden AG, Oostra BA, van Duijn CM. Three genome-wide association studies and a linkage analysis identify HERC2 as a human iris color gene. Am J Hum Genet. 2008 Feb;82(2):411-23. doi: 10.1016/j.ajhg.2007.10.003.](https://www.zotero.org/google-docs/?ONeNiz)

[4. Han J, Kraft P, Nan H, Guo Q, Chen C, Qureshi A, Hankinson SE, Hu FB, Duffy DL, Zhao ZZ, Martin NG, Montgomery GW, Hayward NK, Thomas G, Hoover RN, Chanock S, Hunter DJ. A genome-wide association study identifies novel alleles associated with hair color and skin pigmentation. PLoS Genet. 2008 May 16;4(5):e1000074. doi: 10.1371/journal.pgen.1000074.](https://www.zotero.org/google-docs/?ONeNiz)

[5. Męcińska-Jundziłł K, Tadrowski T, Jundziłł A, Witmanowski H, Czajkowki R. Evaluation of polymorphisms and expression of PTPN22, NLRP1 and TYR genes in vitiligo patients. Postepy Dermatol Alergol. 2023 Apr;40(2):225-233. doi: 10.5114/ada.2023.126314.](https://www.zotero.org/google-docs/?ONeNiz)

[6. Andersen JD, Johansen P, Harder S, Christoffersen SR, Delgado MC, Henriksen ST, Nielsen MM, Sørensen E, Ullum H, Hansen T, Dahl AL, Paulsen RR, Børsting C, Morling N. Genetic analyses of the human eye colours using a novel objective method for eye colour classification. Forensic Sci Int Genet. 2013 Sep;7(5):508-15. doi: 10.1016/j.fsigen.2013.05.003.](https://www.zotero.org/google-docs/?ONeNiz)

[7. Kastelic V, Pośpiech E, Draus-Barini J, Branicki W, Drobnič K. Prediction of eye color in the Slovenian population using the IrisPlex SNPs. Croat Med J. 2013 Aug;54(4):381-6. doi: 10.3325/cmj.2013.54.381.](https://www.zotero.org/google-docs/?ONeNiz)

[8. Kastelic V, Drobnic K. A single-nucleotide polymorphism (SNP) multiplex system: the association of five SNPs with human eye and hair color in the Slovenian population and comparison using a Bayesian network and logistic regression model. Croat Med J. 2012 Oct;53(5):401-8. doi: 10.3325/cmj.2012.53.401.](https://www.zotero.org/google-docs/?ONeNiz)

[9. Jacobs LC, Liu F, Pardo LM, Hofman A, Uitterlinden AG, Kayser M, Nijsten T. IRF4, MC1R and TYR genes are risk factors for actinic keratosis independent of skin color. Hum Mol Genet. 2015 Jun 1;24(11):3296-303. doi: 10.1093/hmg/ddv076.](https://www.zotero.org/google-docs/?ONeNiz)

[10. Rahat MA, Akbar F, Rasool A, Ilyas M, Rakha A, Shams S, Jelani M, Bibi F, Shirah BH, Abdulkareem AA, Naseer MI, Israr M. Phenotypic Classification of Eye Colour and Developmental Validation of the Irisplex System on Population Living in Malakand Division, Pakistan. Biomedicines. 2023 Apr 20;11(4):1228. doi: 10.3390/biomedicines11041228.](https://www.zotero.org/google-docs/?ONeNiz)

[11. Wang Y. Association of pigmentation related-genes polymorphisms and geographic environmental variables in the Chinese population. Hereditas. 2021 Jul 8;158(1):24. doi: 10.1186/s41065-021-00189-7.](https://www.zotero.org/google-docs/?ONeNiz)

[12. Khoruddin NA, Noorizhab MN, Teh LK, Mohd Yusof FZ, Salleh MZ. Pathogenic nsSNPs that increase the risks of cancers among the Orang Asli and Malays. Sci Rep. 2021 Aug 9;11(1):16158. doi: 10.1038/s41598-021-95618-y.](https://www.zotero.org/google-docs/?ONeNiz)

[13. Meyer OS, Lunn MMB, Garcia SL, Kjærbye AB, Morling N, Børsting C, Andersen JD. Association between brown eye colour in rs12913832:GG individuals and SNPs in TYR, TYRP1, and SLC24A4. PLoS One. 2020 Sep 11;15(9):e0239131. doi: 10.1371/journal.pone.0239131.](https://www.zotero.org/google-docs/?ONeNiz)

[14. Reis LB, Bakos RM, Vianna FSL, Macedo GS, Jacovas VC, Ribeiro-Dos-Santos AM, Santos S, Bakos L, Ashton-Prolla P. Skin pigmentation polymorphisms associated with increased risk of melanoma in a case-control sample from southern Brazil. BMC Cancer. 2020 Nov 9;20(1):1069. doi: 10.1186/s12885-020-07485-x.](https://www.zotero.org/google-docs/?ONeNiz)

[15. Potrony M, Rebollo-Morell A, Giménez-Xavier P, Zimmer L, Puig-Butille JA, Tell-Marti G, Sucker A, Badenas C, Carrera C, Malvehy J, Schadendorf D, Puig S. IRF4 rs12203592 functional variant and melanoma survival. Int J Cancer. 2017 Apr 15;140(8):1845-1849. doi: 10.1002/ijc.30605.](https://www.zotero.org/google-docs/?ONeNiz)

[16. Ward SV, Gibbs DC, Orlow I, Thomas NE, Kanetsky PA, Luo L, Cust AE, Anton-Culver H, Gruber SB, Gallagher RP, Rosso S, Zanetti R, Dwyer T, Begg CB, Berwick M; GEM Study Group. Association of IRF4 single-nucleotide polymorphism rs12203592 with melanoma-specific survival. Br J Dermatol. 2020 Jul;183(1):163-165. doi: 10.1111/bjd.18881.](https://www.zotero.org/google-docs/?ONeNiz)

[17. Gibbs DC, Orlow I, Bramson JI, Kanetsky PA, Luo L, Kricker A, Armstrong BK, Anton-Culver H, Gruber SB, Marrett LD, Gallagher RP, Zanetti R, Rosso S, Dwyer T, Sharma A, La Pilla E, From L, Busam KJ, Cust AE, Ollila DW, Begg CB, Berwick M, Thomas NE; GEM Study Group. Association of Interferon Regulatory Factor-4 Polymorphism rs12203592 With Divergent Melanoma Pathways. J Natl Cancer Inst. 2016 Feb 8;108(7):djw004. doi: 10.1093/jnci/djw004.](https://www.zotero.org/google-docs/?ONeNiz)

[18. Wang S, Yan Q, Chen P, Zhao P, Gu A. Association of interferon regulatory factor 4 gene polymorphisms rs12203592 and rs872071 with skin cancer and haematological malignancies susceptibility: a meta-analysis of 19 case-control studies. BMC Cancer. 2014 Jun 6;14:410. doi: 10.1186/1471-2407-14-410.](https://www.zotero.org/google-docs/?ONeNiz)

[19. Peña-Chilet M, Blanquer-Maceiras M, Ibarrola-Villava M, Martinez-Cadenas C, Martin-Gonzalez M, Gomez-Fernandez C, Mayor M, Aviles JA, Lluch A, Ribas G. Genetic variants in PARP1 (rs3219090) and IRF4 (rs12203592) genes associated with melanoma susceptibility in a Spanish population. BMC Cancer. 2013 Mar 27;13:160. doi: 10.1186/1471-2407-13-160.](https://www.zotero.org/google-docs/?ONeNiz)

[20. Onan D, Yorulmaz A, Ezgü FS, Hayran KM, Külcü S, Artüz RF, Yalçın B. The role of IFIH1 gene rs1990760 and rs2111485 single-nucleotide polymorphisms in generalized vitiligo predisposition. Turk J Med Sci. 2019 Feb 11;49(1):206-211. doi: 10.3906/sag-1808-63.](https://www.zotero.org/google-docs/?ONeNiz)

[21. Yao Y, Shen Y, Shao H, Liu Y, Ji Y, Du G, Ye X, Huang P, Chen H. Polymorphisms of RIG-I-like receptor influence HBV clearance in Chinese Han population. J Med Virol. 2021 Aug;93(8):4957-4965. doi: 10.1002/jmv.26969.](https://www.zotero.org/google-docs/?ONeNiz)

[22. Zurawek M, Fichna M, Fichna P, Skowronska B, Dzikiewicz-Krawczyk A, Januszkiewicz D, Nowak J. Cumulative effect of IFIH1 variants and increased gene expression associated with type 1 diabetes. Diabetes Res Clin Pract. 2015 Feb;107(2):259-66. doi: 10.1016/j.diabres.2014.11.008.](https://www.zotero.org/google-docs/?ONeNiz)

[23. Robertson CC, Inshaw JRJ, Onengut-Gumuscu S, Chen WM, Santa Cruz DF, Yang H, Cutler AJ, Crouch DJM, Farber E, Bridges SL Jr, Edberg JC, Kimberly RP, Buckner JH, Deloukas P, Divers J, Dabelea D, Lawrence JM, Marcovina S, Shah AS, Greenbaum CJ, Atkinson MA, Gregersen PK, Oksenberg JR, Pociot F, Rewers MJ, Steck AK, Dunger DB; Type 1 Diabetes Genetics Consortium; Wicker LS, Concannon P, Todd JA, Rich SS. Fine-mapping, trans-ancestral and genomic analyses identify causal variants, cells, genes and drug targets for type 1 diabetes. Nat Genet. 2021 Jul;53(7):962-971. doi: 10.1038/s41588-021-00880-5.](https://www.zotero.org/google-docs/?ONeNiz)

[24. Zhang M, Qureshi AA, Guo Q, Han J. Genetic variation in DNA repair pathway genes and melanoma risk. DNA Repair (Amst). 2011 Jan 2;10(1):111-6. doi: 10.1016/j.dnarep.2010.08.005.](https://www.zotero.org/google-docs/?ONeNiz)

[25. Tang XF, Zhang Z, Hu DY, Xu AE, Zhou HS, Sun LD, Gao M, Gao TW, Gao XH, Chen HD, Xie HF, Tu CX, Hao F, Wu RN, Zhang FR, Liang L, Pu XM, Zhang JZ, Han JW, Pan GP, Wu JQ, Li K, Su MW, Du WD, Zhang WJ, Liu JJ, Xiang LH, Yang S, Zhou YW, Zhang XJ. Association analyses identify three susceptibility Loci for vitiligo in the Chinese Han population. J Invest Dermatol. 2013 Feb;133(2):403-10. doi: 10.1038/jid.2012.320.](https://www.zotero.org/google-docs/?ONeNiz)

[26. Espino-Paisan L, de la Calle H, Fernández-Arquero M, Figueredo MA, de la Concha EG, Urcelay E, Santiago JL. Polymorphisms in chromosome region 12q13 and their influence on age at onset of type 1 diabetes. Diabetologia. 2011 Aug;54(8):2033-7. doi: 10.1007/s00125-011-2177-6.](https://www.zotero.org/google-docs/?ONeNiz)

[27. Johnson K, Wong R, Barriga KJ, Klingensmith G, Ziegler AG, Rewers MJ, Steck AK. rs11203203 is associated with type 1 diabetes risk in population pre-screened for high-risk HLA-DR,DQ genotypes. Pediatr Diabetes. 2012 Dec;13(8):611-5. doi: 10.1111/j.1399-5448.2012.00888.x.](https://www.zotero.org/google-docs/?ONeNiz)

[28. Steck AK, Dong F, Wong R, Fouts A, Liu E, Romanos J, Wijmenga C, Norris JM, Rewers MJ. Improving prediction of type 1 diabetes by testing non-HLA genetic variants in addition to HLA markers. Pediatr Diabetes. 2014 Aug;15(5):355-62. doi: 10.1111/pedi.12092.](https://www.zotero.org/google-docs/?ONeNiz)

[29. Yang XK, Liu J, Chen SY, Li M, Zhang MM, Leng RX, Pan HF, Shen Y, Liu WX, Xu SQ, Ye DQ, Shuai ZW. UBASH3A gene polymorphisms and expression profile in rheumatoid arthritis. Autoimmunity. 2019 Feb;52(1):21-26. doi: 10.1080/08916934.2019.1581773.](https://www.zotero.org/google-docs/?ONeNiz)

[30. Newman JRB, Concannon P, Ge Y. UBASH3A Interacts with PTPN22 to Regulate IL2 Expression and Risk for Type 1 Diabetes. Int J Mol Sci. 2023 May 12;24(10):8671. doi: 10.3390/ijms24108671.](https://www.zotero.org/google-docs/?ONeNiz)

[31. Zhang XH, Shen M, Liu L, Li FM, Hu PC, Hua Q, Zhang J, Pang LN, Lu HW, Wang ZM, Chu X, Huang W. Association Analysis of Single Nucleotide Polymorphisms in C1QTNF6, RAC2, and an Intergenic Region at 14q32.2 with Graves’ Disease in Chinese Han Population. Genet Test Mol Biomarkers. 2017 Aug;21(8):479-484. doi: 10.1089/gtmb.2017.0009.](https://www.zotero.org/google-docs/?ONeNiz)

[32. Li Q, Wang B, Pan F, Zhang R, Xiao L, Guo H, Ma S, Zhou C. Association between cytotoxic T-lymphocyte antigen 4 gene polymorphisms and primary biliary cirrhosis in Chinese population: data from a multicenter study. J Gastroenterol Hepatol. 2013 Aug;28(8):1397-402. doi: 10.1111/jgh.12165.](https://www.zotero.org/google-docs/?ONeNiz)

[33. Yang XC, Fujino M, Cai SJ, Li SW, Liu C, Li XK. Genetic Polymorphisms of Cytotoxic T-Lymphocyte Antigen 4 in Primary Biliary Cholangitis: A Meta-Analysis. J Immunol Res. 2017;2017:5295164. doi: 10.1155/2017/5295164. Epub 2017 May 31.](https://www.zotero.org/google-docs/?ONeNiz)

[34. Joshita S, Umemura T, Yoshizawa K, Katsuyama Y, Tanaka E, Nakamura M, Ishibashi H, Ota M; Shinshu PBC Study Group. Association analysis of cytotoxic T-lymphocyte antigen 4 gene polymorphisms with primary biliary cirrhosis in Japanese patients. J Hepatol. 2010 Sep;53(3):537-41. doi: 10.1016/j.jhep.2010.03.017.](https://www.zotero.org/google-docs/?ONeNiz)

[35. Aiba Y, Nakamura M, Joshita S, Inamine T, Komori A, Yoshizawa K, Umemura T, Horie H, Migita K, Yatsuhashi H, Nakamuta M, Fukushima N, Saoshiro T, Hayashi S, Kouno H, Ota H, Muro T, Watanabe Y, Nakamura Y, Komeda T, Shimada M, Masaki N, Komatsu T, Yagura M, Sugi K, Koga M, Tsukamoto K, Tanaka E, Ishibashi H; PBC Study Group in NHOSLJ. Genetic polymorphisms in CTLA4 and SLC4A2 are differentially associated with the pathogenesis of primary biliary cirrhosis in Japanese patients. J Gastroenterol. 2011 Oct;46(10):1203-12. doi: 10.1007/s00535-011-0417-7.](https://www.zotero.org/google-docs/?ONeNiz)

[36. Li M, Zheng H, Li T, Gao P, Zhang XL, Liu DW. Cytotoxic T-lymphocyte associated antigen-4 gene polymorphisms and primary biliary cirrhosis: a systematic review. J Gastroenterol Hepatol. 2012 Jul;27(7):1159-66. doi: 10.1111/j.1440-1746.2012.07118.x.](https://www.zotero.org/google-docs/?ONeNiz)

[37. Deng L, Zhou H, Yang J, Xiao J, Wang B, Wang L, Ou X, Feng Y. CTLA-4 gene polymorphisms and susceptibility to chronic obstructive pulmonary disease. Int J Clin Exp Pathol. 2013 Oct 15;6(11):2548-53.](https://www.zotero.org/google-docs/?ONeNiz)

[38. Song SH, Wang XQ, Shen Y, Hong SL, Ke X. Association between PTPN22/CTLA-4 Gene Polymorphism and Allergic Rhinitis with Asthma in Children. Iran J Allergy Asthma Immunol. 2016 Oct;15(5):413-419.](https://www.zotero.org/google-docs/?ONeNiz)

[39. Yan F, Gao YF, Lv F, Zhang TC, Li X, Yin HF. No association between IRF3 polymorphism and susceptibility to hepatitis B virus infection in Chinese patients. World J Gastroenterol. 2012 Jan 28;18(4):388-92. doi: 10.3748/wjg.v18.i4.388.](https://www.zotero.org/google-docs/?ONeNiz)

[40. Santana-de Anda K, Gómez-Martín D, Monsivais-Urenda AE, Salgado-Bustamante M, González-Amaro R, Alcocer-Varela J. Interferon regulatory factor 3 as key element of the interferon signature in plasmacytoid dendritic cells from systemic lupus erythematosus patients: novel genetic associations in the Mexican mestizo population. Clin Exp Immunol. 2014 Dec;178(3):428-37. doi: 10.1111/cei.12429.](https://www.zotero.org/google-docs/?ONeNiz)

[41. Wang H, Jin Y, Reddy MV, Podolsky R, Liu S, Yang P, Bode B, Reed JC, Steed RD, Anderson SW, Steed L, Hopkins D, Huang Y, She JX. Genetically dependent ERBB3 expression modulates antigen presenting cell function and type 1 diabetes risk. PLoS One. 2010 Jul 26;5(7):e11789. doi: 10.1371/journal.pone.0011789.](https://www.zotero.org/google-docs/?ONeNiz)
