# Supplementary figures and images for: Estimation of genetic variation in vitiligo associated genes: Population genomics perspective
Source: BMC Genom Data. 2024 Jul 26;25:72. doi: 10.1186/s12863-024-01254-6 (PMC11282599; doi:10.1186/s12863-024-01254-6)

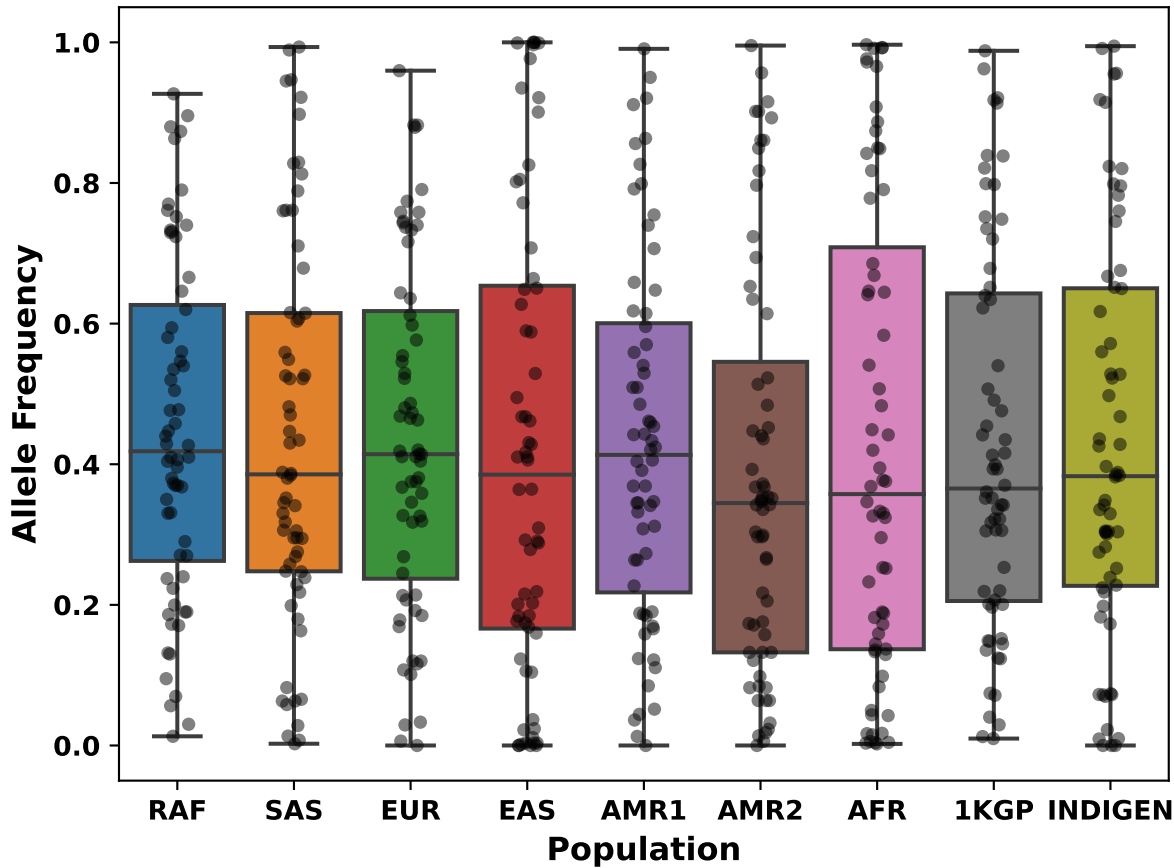

Supplement: Supplementary file 2 — Supplementary Figure 01: Comparison of vitiligo associated genetic risk scores from GWAS across super-populations reported in 1000 Genome and IndiGenomes Project [file 12863_2024_1254_MOESM2_ESM.pdf]

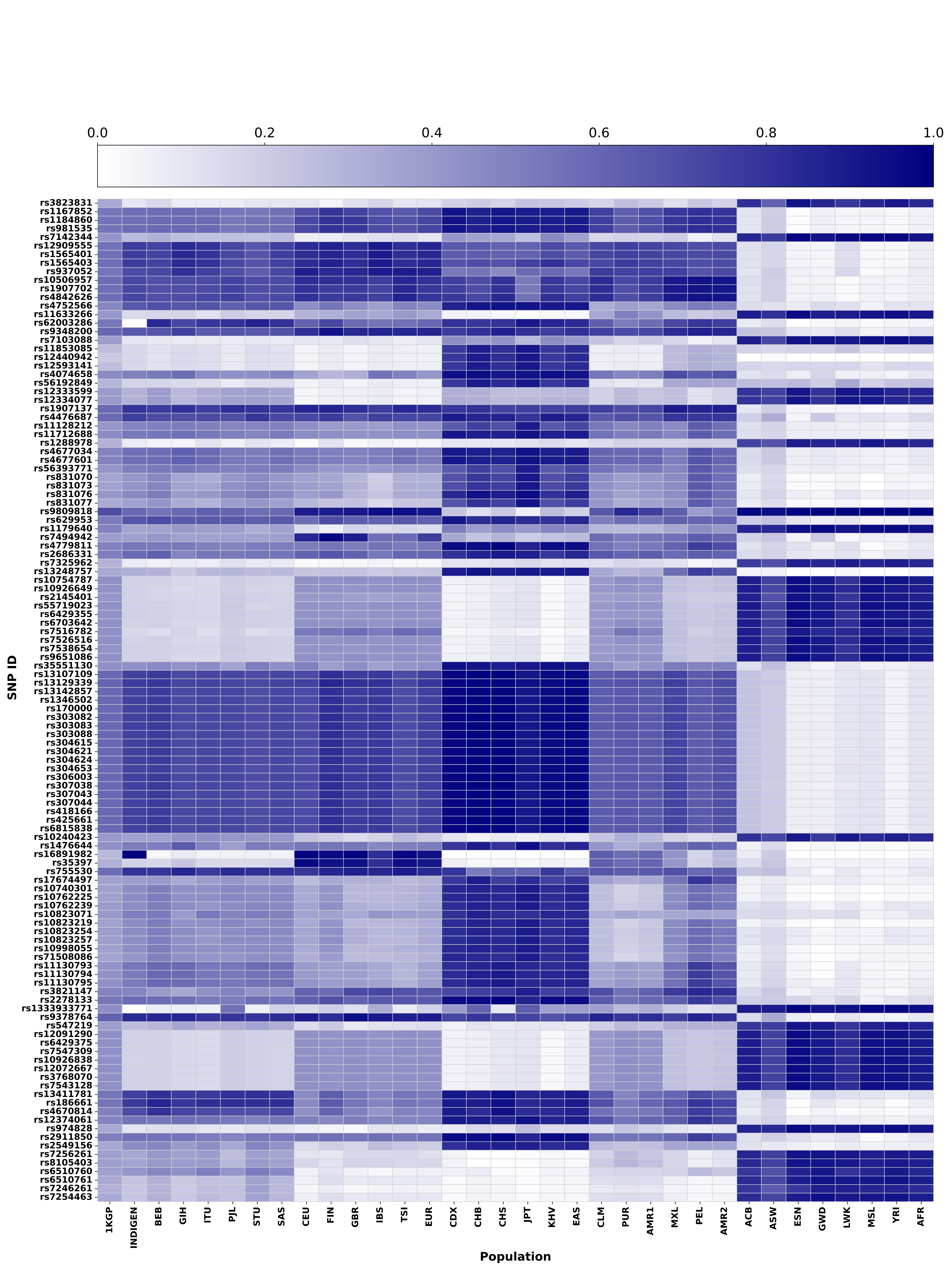

Supplement: Supplementary file 3 — Supplementary Figure 02: Distribution of vitiligo associated SNP frequency from prominent databases and literature across super-populations / populations reported in 1000 Genome and IndiGenomes project [file 12863_2024_1254_MOESM3_ESM.pdf]

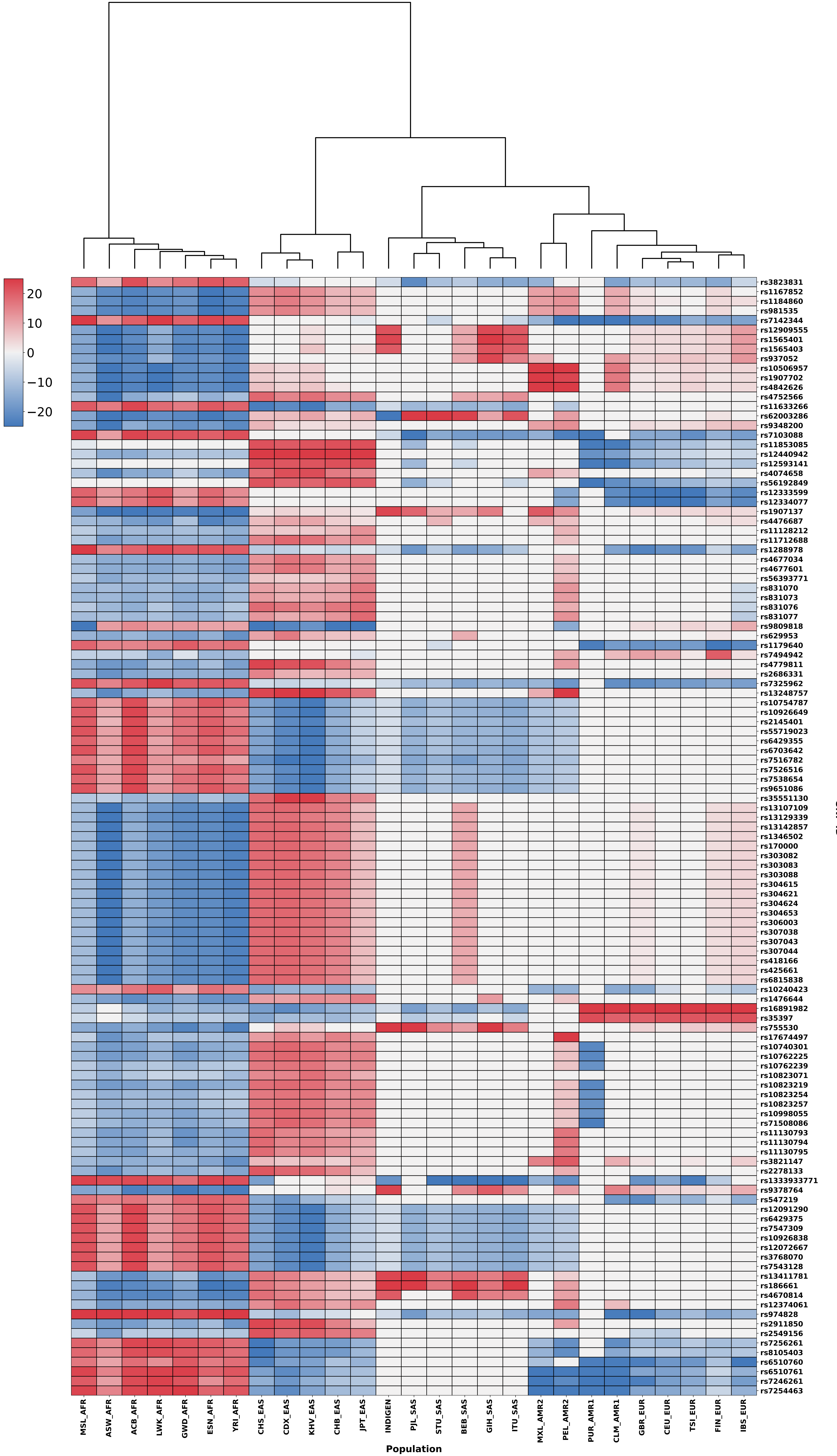

Supplement: Supplementary file 4 — Supplementary Figure 03: Enriched and depleted pattern of significant variation in 117 SNPs of vitiligo in across populations reported in 1000 Genome and IndiGenomes project [file 12863_2024_1254_MOESM4_ESM.pdf]
